# Hospitalization due to pneumococcal disease in the Unified Health System in Brazil: A retrospective analysis of administrative data

**DOI:** 10.1016/j.bjid.2024.104482

**Published:** 2024-11-27

**Authors:** Daniela V. Pachito, Mauricio Longato, Guilherme Cordeiro, Paulo H.R.F. Almeida, Ricardo Macarini Ferreira, Ana Paula N. Burian

**Affiliations:** a Pfizer Brazil, São Paulo, SP, Brazil; b Analytix, São Paulo, SP, Brazil; c Secretaria Estadual de Saúde do Espírito Santo, Vitória, ES, Brazil

**Keywords:** Pneumococcal infections, Health care economics and organizations, Costs and cost analysis, Unified health system

## Abstract

**Introduction:**

Community-Acquired Pneumonia (CAP) caused by pneumococcus and Invasive Pneumococcal Disease (IPD) pose a substantial economic burden on health systems. The objective of the present study is to explore hospitalization costs of pneumococcal disease in the public health system in Brazil, the Unified Health System.

**Methods:**

Retrospective analysis of administrative data on hospitalized cases of pneumococcal disease from January 2019 to July 2023. Hospitalization cases recorded with ICD-10 codes of CAP due to *S. pneumoniae* and IPD were retrieved from DATASUS, the inpatient information system of the Unified Health System in Brazil. Costs were converted to US dollars by Using Purchasing Power Parity (USD-PPP). Absolute number of hospitalizations, costs of hospitalizations and healthcare resource utilization were presented descriptively. The annual cost estimate was calculated. Differences in costs by type of clinical presentation and age group were assessed. Factors associated with higher costs were explored by multiple linear regression models.

**Results:**

A total of 22,498 hospitalization episodes were analyzed. Total cost of hospitalizations was USD-PPP 13,958,959 (BRL 34,659,578) with an annual mean estimate of USD-PPP 3,045,591 (BRL 7,562,090). Cost per hospitalization episode was significantly higher for meningitis, followed by septicemia, CAP and arthritis, with median values ranging from USD-PPP 190.93 to 615.14 (BRL 476.20 to 1529.02). (Kruskal-Wallis *χ*^2^ = 6473, *df* = 3, *p*-value < 0.0001). Costs were significantly higher among individuals aged 60-years and older. (Kruskal-Wallis test; *χ*^2^ = 773.53; *df* = 2, *p*-value < 0.0001). There were differences in age at hospitalization, length of stay, and ICU utilization among types of clinical presentations.

**Conclusions:**

Our findings reveal the economic burden associated with pneumococcal disease in the Unified Health System in Brazil. Hospitalization costs were higher for cases of meningitis and among individuals aged 60-years and above.

## Introduction

*Streptococcus pneumoniae* or Pneumococcus is the leading cause of Community-Acquired Pneumonia (CAP), accounting for 20–60 % of cases.^1^, ^2^, ^3^, ^4^ Pneumococcus is also associated with clinical presentations marked by the contamination of normally sterile specimens, in which cases the disease is called Invasive Pneumococcal Disease. (IPD).^5^ Altogether, this group of diseases requires specialized healthcare resources, ultimately posing a high economic burden on health systems. From the humanistic perspective, CAP and IPD are associated with a high morbidity and mortality, especially among vulnerable populations, such as individuals with comorbidities, young children, and the elderly, and in cases of infection by more virulent serotypes.^6^, ^7^, ^8^

Pneumococcal conjugate vaccines and pneumococcal polysaccharide vaccines have been implemented worldwide as an effective strategy to reduce the burden of disease.^9^^,^^10^ However, optimization of vaccination depends on factors such as the immunization coverage and the dynamics of circulating serotypes of pneumococcus. The predominance of non-vaccine serotypes observed after the institution of the immunization program, also known as serotype replacement, challenges the long-term effectiveness of immunization strategies, imposing the need for the adoption of schemes with a broader coverage and sustained immune responses.^11^ This may help explain why pneumococcal disease remains a public health issue despite many advances regarding the immunization strategies.^12^

Patterns of utilization of healthcare resources due to pneumococcal disease have been investigated in several studies in recent years. In the study conducted by Mohanty et al. in the USA, over 80,000 hospitalization episodes of adults with ICD-10 codes of pneumococcal disease and/or positive culture tests were analyzed. The vast majority of cases consisted of non-invasive CAP (98.6 %), with a median Length of Stay (LOS) of 6 days and median hospitalization cost of USD 9,746. For IPD cases, the observed LOS was 8 days at a cost of $13,194, with negative margins (total payments to hospitals minus total costs). However, these findings cannot be extrapolated to other settings since healthcare costs are highly variable across settings and countries. In a study conducted in a university hospital in Brazil, 186 cases of CAP caused by *S. pneumoniae* were analyzed. The mean length of stay for individuals under 65 years old was 10 days, with a mean hospitalization cost of USD 1,515, and 14 days for aged individuals, with mean hospitalization cost of USD 1,902.^13^

The objective of the present study is to present the number of hospitalization episodes due to IPD and pneumonia caused by *S. pneumoniae* in the Unified Health System in Brazil, as well as to estimate the cost of hospitalization episodes and the length of stay and explore factors associated with higher costs.

## Methods

### Study design and study population

A retrospective analysis of administrative data on hospitalizations due to pneumococcal diseases from 01 January 2019 to 31 July 2023. Clinical presentation of pneumococcal diseases considered in this study were IPD (namely septicemia, meningitis, and septic arthritis), and CAP.

The study population consisted of individuals, regardless of sex and age, with at least one episode of hospitalization attributed to one of the ICD-10 codes presented in Table 1. Besides the four types of pneumococcal disease of interest, the inclusion of the disease-agnostic code B95.3 (*Streptococcus pneumoniae* as the cause of diseases classified to other chapters) was adopted to improve the identification of hospitalization cases due to pneumococcal infection.

**Table 1:** ICD-10 codes applied in analyses.

Invasive disease (septicemia, meningitis, arthritis) due to *S. pneumoniae*	
A40.3	Septicemia due to *S. pneumoniae*
G00.1	Pneumococcal meningitis
M00.1	Pneumococcal arthritis and polyarthritis
Non-invasive disease (pneumonia) *due to Streptococcus pneumoniae*	
J13	Pneumonia due to Streptococcus pneumoniae
Disease-agnostic code for pneumococcal infection	
B95.3	Streptococcus pneumoniae as the cause of diseases classified to other chapters

ICD-10, International Classification of Diseases 10th Edition.

### Study setting and data sources

The Unified Health System (*Sistema Único de Saúde* or SUS) is currently the exclusive source of health care for roughly three-quarters of the Brazilian population, thus being considered one of the largest universal health systems globally.^14^ The Department of Informatics of the Unified Health System (*Departamento de Informática do Sistema Único de Saúde* or DATASUS) aggregates a myriad of Information Systems that contain data on births, Health Care Resource Utilization (HCRU) at the medium and high complexity levels of healthcare, vaccination, and mortality, among other information.^15^ Inpatient HCRU in SUS is recorded in the Inpatient Information System (*Sistema de Informação Hospitalar* or SIH). SIH is essentially an administrative database developed for the reimbursement of procedures for the healthcare providers that are part of the SUS.^16^ The SIH retains information on demographics (sex and age), clinical data, such as main and secondary diagnosis, length of stay, days in the Intensive Care Unit (ICU), performed procedures, and paid amounts.^16^

### Data extraction and cleaning

Data from SIH was extracted from the URL publicized by the Brazilian Ministry of Health. From the table named RD, data on birth date, hospitalization date, ICD-10 codes, length of stay, length of stay in ICU, and cost of the hospitalization episode were retrieved and combined into a single dataset. Age at hospitalization was calculated by subtracting the birth date from the hospitalization date. For comparative analysis, cases were grouped into three age groups, namely (i) Children 5-years old and under; (ii) An intermediate age group; and (iii) Individuals aged 60-years and older.

During the process of data cleaning, observations with length of stay attributed to 0, or for which main ICD-10 codes were different from the ICD-10 codes of interest, or with no attributed hospitalization cost were excluded from analyses. No type of data imputation was performed.

### Currency exchange rates

We converted cost estimates to American dollars (USD) by applying Purchasing Power Parity (PPP) as per the methods applied by the Organization for Economic Co-operation and Development.^17^ The rationale for choosing USD-PPP rates instead of commercial currency exchange rates was based on the fact that USD-PPP rates have an annual periodicity and provide a standard measure that allows for comparisons between countries.^18^

### Statistical analyses

The absolute number of hospitalization episodes assigned with the ICD-10 codes of interest was presented, along with the measures of central tendency (mean, median) and dispersion (standard deviation and 1st and 3rd quartiles) for hospitalization costs and HCRU (e.g., length of stay, days in the intensive unit care). Mean annual cost was calculated by dividing the total cost of hospitalization by the number of years in the period of analysis.

Normality was assessed with the Kolmogorov-Smirnov test. Since variables of interest did not follow a parametric distribution, comparative analyses were conducted with the Kruskal-Wallis test. Factors associated with higher costs were explored by multiple linear regression models. Analyses were conducted using packages ggplot2 version 3.4.2, gridExtra version 2.3 and dplyr version 1.1.2 in R version 4.1.0.

### Ethical approval

The analyses were limited to publicly available deidentified data, resulting in no need for an Institutional Research Board review.

## Results

### Hospitalization episodes

A total of 23,661 observations were retrieved for the period of analysis of 4.6 years. Observations with the value of length of stay attributed to 0 were removed (*n* = 451). Subsequently, observations for which the main ICD-10 code differed from those of interest (i.e., ICD-10 codes A403; G001; M001; J13; and B95.3) were removed (*n* = 711). Finally, one observation with no value attributed to hospitalization cost was removed, reaching the final dataset composed of 22,498 hospitalization episodes. Of note, no hospitalization assigned with the ICD-10 code B95.3 (*Streptococcus pneumoniae as the cause of diseases classified to other chapters*) was retrieved. Altogether, the hospitalization episodes summed a total cost of USD-PPP 13,958,959 (BRL 34,659,578), with an annual mean estimate of USD-PPP 3,045,591 (BRL 7,567,593).

**Fig. 1:**
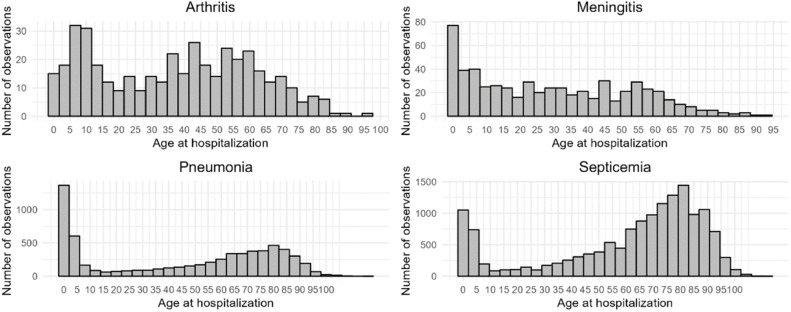
Age at hospitalization by clinical presentation.

Characterization of cases by sex and age and the proportion of cases by disease type are presented in Table 2. There was a predominance of male sex for all disease categories. Age at hospitalization was significantly different across disease categories, with younger ages at hospitalization being observed for meningitis and arthritis, followed by pneumonia and septicemia (Fig. 1). For all four clinical presentations, a bimodal distribution of cases was found, with the first peak occurring during childhood and a second one in late middle age or old age.

**Table 2:** Characterization of cases by sex and age, according to clinical presentation.

	Male (%)	Age (mean, SD)	Proportion of cases
Septicemia; ICD-10 A403 (*n* = 14,846)	7664 (51.6 %)	60.6 ± 28.9	65.9 %
Meningitis; ICD-10 G001 (*n* = 587)	324 (55.2 %)	29.5 ± 23.3	2.6 %
Pneumococcal arthritis and polyarthritis; ICD-10 M001 (*n* = 419)	281 (67.1 %)	37.2 ± 23.7	1.9 %
Pneumonia due to Streptococcus pneumoniae; ICD-J13 (*n* = 6646)	3449 (51.9 %)	45.4 ± 34.2	29.6 %
Total sample (*n* = 22,498)	11,719 (52 %)	54.9 ± 31.5	100 %

ICD-10, International Classification of Diseases 10th Edition; SD, Standard Deviation.

Comparative analysis of costs across age groups, namely (i) Children under 5-years-old; (ii) Intermediate age group; and (iii) Individuals aged 60-years and above, showed higher costs for individuals aged 60-years and above. (Kruskal-Wallis test; *χ*^2^ = 773.53; *df* = 2, *p*-value < 0.0001).

Cost estimates in Brazilian Real (BRL) are presented in the Supplemental Table 1. PPP rates for converting BRL values into USD-PPP are presented in Supplemental Table 2.

### Health care resource utilization

**Fig. 2:**
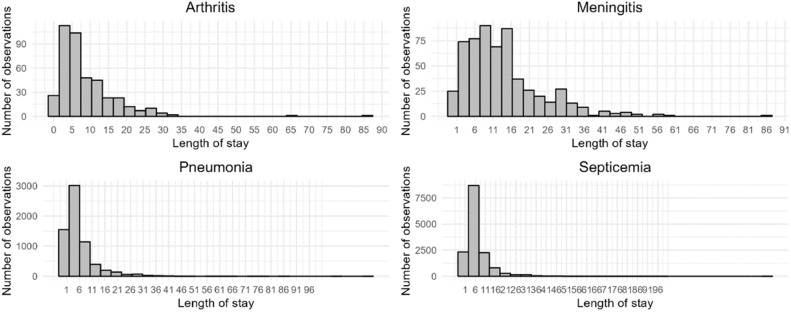
Length of stay by clinical presentation.

Mean length of stay in the hospital and in the ICU by disease type is presented in Fig. 2 and Table 3. There were statistically significant differences in the length of stay, with longer stays for the cases of meningitis, followed by arthritis, septicemia and CAP (Kruskal-Wallis *χ*^2^ = 545.31, *df* = 3, *p*-value < 0.0001). Across all clinical presentations, few cases required hospitalization in the ICU, and meningitis was the clinical presentation associated with a greater utilization of ICU.

**Table 3:** Costs and resource utilization in the total sample and by clinical presentation.

		Min	Median (IQR)	Max
Total sample (*n* = 22,498)	Hospitalization cost (USD-PPP)	16	361 (121)	32,024
	Length of stay (days)	1	5 (5)	151
	Length of stay in the ICU (days)	0	0 (0)	121
Septicemia ICD-10 A403 (*n* = 14,846)	Hospitalization cost (USD-PPP)	17	368 (54)	32,024
	Length of stay (days)	1	5 (5)	151
	Length of stay in the ICU (days)	0	0 (0)	92
Meningitis ICD-10 G001 (*n* = 587)	Hospitalization cost (USD-PPP)	19	615 (1515)	15,307
	Length of stay (days)	1	12	87
	Length of stay in the ICU (days)	0	0 (5)	34
Pneumococcal arthritis and polyarthritis ICD-10 M001 (*n* = 419)	Hospitalization cost (USD-PPP)	16	191 (260)	7011
	Length of stay (days)	1	7 (9)	87
	Length of stay in the ICU (days)	0	0 (0)	19
Pneumonia due to Streptococcus pneumoniae ICD-J13 (*n* = 6646)	Hospitalization cost (USD-PPP)	17	249 (56)	30,069
	Length of stay (days)	1	4 (4)	121
	Length of stay in the ICU (days)	0	0 (0)	121

ICD-10, International Classification of Diseases 10th Edition; IQR, Interquartile Range; Max, Maximum; Min, Minimum; USD-PPP, US Dollar converted by purchasing power parity rates.

### Hospitalization costs

**Fig. 3:**
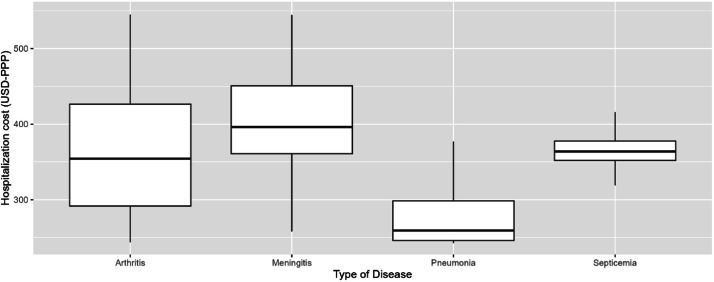
Hospitalization costs by clinical presentation.

Hospitalization costs were significantly higher for meningitis, followed by septicemia, CAP and arthritis (Table 3, Fig. 3) (Kruskal-Wallis *χ*^2^ = 6473, *df* = 3, *p*-value < 0.00001).

There was a statistically significant difference of hospitalization costs across age groups, with higher costs being observed among individuals aged 60 years or older. Multiple linear regression showed that the clinical presentation influenced hospitalizations costs, with no influence of sex or age, as a continuous variable (Adjusted R2: 0.02827; F-statistic: 131.9 on 5 and 22,492 DF, *p*-value < 0.0001, Supplemental Table 3).

## Discussion

Our study assessed the economic burden of hospitalizations due to pneumococcal disease in the Unified Health System by analyzing data from DATASUS. Despite the existing immunization program, 22,498 hospitalization episodes with the ICD-10 codes related to pneumococcal disease were retrieved in the period of analysis.

Regarding the types of clinical presentation, the most prevalent cases were septicemia (65.9 %), followed by CAP (29.6 %), meningitis (2.6 %), and arthritis (1.9 %). In the study conducted by Morrill et al. in the U.S., the proportion of clinical presentations were markedly different, with a predominance of non-invasive pneumonia (63.4 %).^19^ In the study conducted in Brazil by Medeiros et al., the proportion of cases of pneumonia among individuals hospitalized with pneumococcal disease in a tertiary Brazilian hospital was 67.8 %, which is closer to the proportion reported by Morril et al. and it is much higher than what was observed in our study (i.e., 29.6 % of cases).^20^ One explanation for such a discrepancy is the possibility of coding errors or the use of unspecified ICD-10 codes in our study (e.g., J15: bacterial pneumonia, not elsewhere classified), which may have led to the underestimation of the number of cases of CAP caused by pneumococcus.

Another explanation for the relatively low proportion of cases of CAP in our study may relates to the need of instituting empiric therapy. In a cohort study in Brazil, blood or sputum cultures were collected in only 33 % of hospitalized patients with CAP, with roughly one-half of the cultures isolating no pathogen.^21^ The standard for aetiological investigation of CAP in Brazil remains blood culture, which may take up to 72 h to produce results.^22^ The high proportion of cases of CAP with undetermined aetiology was additionally confirmed by other studies.^23^^,^^24^

Another study conducted in three health facilities in Brazil reported a predominance of pneumococcal meningitis, representing 78.1 % of cases, which is not corroborated by our results. Authors concluded that this finding was explained by the vigilance strategies in place, which imposes the notification of meningitis cases and the routine of performing blood and cerebrospinal fluid cultures.^25^

For all clinical presentations, there was a slightly predominance of cases among males. This predominance has been reported in previous studies. In a population-based prospective cohort study conducted in Spain, incidence rates of CAP caused by S. *pneumoniae* were statistically higher in men in all age groups, being more than double among individuals aged 75 years and older.^26^ The impact of the use of pneumococcal conjugate vaccines on these differences is still controversial. In a study conducted in the United States, incidence rates of IPD after the implementation of a pneumococcal conjugate vaccine remained significantly higher among males of certain age groups.^27^ However, patterns of serotype replacement after immunization programs has been show to differ according to sex and age groups, which brings complexity to the understanding of the infection dynamics over time.^28^

In our study, cases followed a bi-modal pattern of incidence, with a first peak occurring in children and a second one occurring in later stages of life. For meningitis and pneumonia, the first peak comprised most cases, with septicemia and arthritis cases peaking more expressively in late middle age or being more evenly distributed across the lifespan. This finding is corroborated by a previous study conducted in Canada, in which the highest incidence rates of confirmed IPD cases were observed among children aged 0‒4 years, followed by individuals aged 65 years and older.^29^

Hospitalization costs differed across types of disease and age groups. Meningitis was the clinical presentation associated with higher hospitalization costs, length of hospital stay and utilization of ICU. Findings of a systematic review indicated that hospitalization costs per patient due to pneumococcal meningitis vary from USD-PPP 5,120 in South Korea to USD-PPP 47,473 in the USA.^30^ In our study, median hospitalization cost of cases with pneumococcal meningitis was USD-PPP 615.14 (BRL 1529.02), which is far below the value observed in other countries. This discrepancy may be explained by factors such as differences in the patterns of utilization of healthcare resources across different settings and economies and in the valuation of such resources, the precision of the costing methodology applied in studies (i.e., gross costing vs micro-costing), and issues of underfunding in the Unified Health System.^31^ It is also possible that costs had not been accurately recorded in DATASUS. Minimum values of hospitalization costs for all clinical presentations were below USD-PPP-20, raising the possibility of entry errors. Although we recognize that these values are unrealistic, we only excluded cases with no value attributed to cost to avoid setting an arbitrary minimum threshold.

Hospitalization episodes among individuals aged 60 years and above were found to be more costly. This finding is corroborated by the study conducted by Michelin et al. in Brazil that investigated differences in hospitalization costs due to pneumococcal pneumonia among patients aged 65 years and older, in comparison with younger patients. Authors reported higher direct hospitalization costs for the older population, with the mean value of USD 1,902. It is noteworthy that the hospitalization costs observed in this study were much higher than the hospitalization cost of cases with CAP in our study, in which the median value was USD-PPP 249.39 (BRL 614.42), which may be explained by the type of setting, considering that the setting studied by Michelin was a highly specialized University hospital.

The main strength of the present study refers to the comprehensiveness of the data source. Instead of presenting data for a given hospital or a set of hospitals, we explored the entire number of hospitalization cases in the public health system in Brazil, warranting the representativeness of findings from the perspective of the Unified Health System.

This study possesses a few limitations. The first limitation refers to the accuracy of the ICD-10 codes as recorded in DATASUS. The possibility of coding errors (i.e., assigning wrong or unspecified ICD-10 codes for cases or attributing codes of pneumococcal disease for non-cases) were anticipated. Of special concern is the possibility of the widespread use of unspecified ICD-10 codes for pneumonia or for the other clinical presentations, leading to an understimation of the number of hospitalizations and the economic burden of pneumococcal disease. In a study assessing referrals to specialized care in Brazil, in roughly two-thirds of records, the unspecified ICD-10 code Z72.9, “Problem related to lifestyle, unspecified” was used, which suggests the widespread use of such codes in certain settings.^32^ Secondly, the nature of the data source (i.e., administrative data rather than medical records) imposes restrictions in terms of the availability of clinical information. No information on comorbidities or serotypes were available, nor the distinction between invasive and non-invasive pneumonia could be made, which would be valuable for understanding the drivers for a more intense utilization of resources and higher costs, in terms of the patient health condition and virulence of pathogens.

Our findings bring important implications from the perspective of the health system. The identification of age groups at higher risk for hospitalization due to pneumococcal disease may guide targeted preventive strategies. Furthermore, the understanding of the volume and distribution of cases are crutial for an optimal resource allocation. Future research is warranted to investigate the impact of ICD-10 coding errors on the estimates of the number of cases and the magnitude of the economic burden herein presented.

## Conclusions

Hospitalization episodes due to pneumococcal disease pose a high economic burden in the Unified Health System. Hospitalization costs varied across clinical presentations and age groups, with higher values being observed for cases of meningitis and among individuals aged 60 years and above. These findings should be considered in the planning and design of health programs directed to pneumococcal disease.

## Conflicts of interest

DVP, PHRFA, and RMF are employees at Pfizer Brazil. ML and GC are paid consultants to Pfizer in connection with the development of this manuscript. APNB is a paid consultant to Pfizer in connection with the development of this manuscript and other projects.

## Appendix Supplementary materials

Image, application 1
