# Supplementary material for: Hospitalization due to pneumococcal disease in the Unified Health System in Brazil: A retrospective analysis of administrative data
Source: Braz J Infect Dis. 2024 Nov 27;29(1):104482. doi: 10.1016/j.bjid.2024.104482 (PMC11638580; doi:10.1016/j.bjid.2024.104482)
Supplement: Supplementary file 1 [file mmc1.docx]

BJID-D-24-00178_Supplementary Material

**Supplemental Table 1** Costs and resource utilization in the total sample and by clinical presentation, in BRL

|  |  | **Min** | **1^st^Qu.** | **Median** | **Mean** | **SD** | **3^rd^ Qu.** | **Max** |
| --- | --- | --- | --- | --- | --- | --- | --- | --- |
| **Total sample (n = 22,498)** | Hospitalization cost (BRL) | 40.38 | 674.5 | 889.9 | 1540.5 | 2899.61 | 970.1 | 82001.1 |
|  | Length of stay (days) | 1 | 3 | 5 | 6.8 | 6.95 | 8 | 151 |
|  | Length of stay in the ICU (days) | 0 | 0 | 0 | 0.97 | 4.07 | 0 | 121 |
| **Septicemia ICD-10 A403 (n = 14,846)** | Hospitalization cost (BRL) | 44.22 | 873.91 | 897.91 | 1658.05 | 2962.65 | 1002.32 | 82001.05 |
|  | Length of stay (days) | 1 | 3 | 5 | 6.66 | 6.68 | 8 | 151 |
|  | Length of stay in the ICU (days) | 0 | 0 | 0 | 1.04 | 4.15 | 0 | 92 |
| **Meningitis ICD-10 G001 (n = 587)** | Hospitalization cost (BRL) | 45.93 | 970.70 | 1529.02 | 4046.99 | 5321.76 | 4844.94 | 39194.80 |
|  | Length of stay (days) | 1 | 7 | 12 | 13.97 | 10.8 | 18 | 87 |
|  | Length of stay in the ICU (days) | 0 | 0 | 0 | 3.92 | 7.32 | 5 | 34 |
| **Pneumococcal arthritis and polyarthritis ICD-10 M001 (n = 419)** | Hospitalization cost (BRL) | 40.38 | 246.70 | 476.20 | 971.58 | 1698.12 | 896.17 | 17952.15 |
|  | Length of stay (days) | 1 | 3 | 7 | 8.86 | 8.34 | 12 | 87 |
|  | Length of stay in the ICU (days) | 0 | 0 | 0 | 0.44 | 1.96 | 0 | 19 |
| **Pneumonia due to Streptococcus pneumonia ICD-J13 (n = 6,646)** | Hospitalization cost (BRL) | 40.38 | 582.42 | 614.42 | 1092.60 | 2317.43 | 727.32 | 76993.96 |
|  | Length of stay (days) | 1 | 3 | 4 | 6.36 | 6.64 | 7 | 121 |
|  | Length of stay in the ICU (days) | 0 | 0 | 0 | 0.59 | 3.43 | 0 | 121 |

**Supplemental Table 2** Purchase Power Parity (PPP) exchange rates from Brazilian reais to US dollars, 2019‒2023.

| **Year** | **Mean PPP rate** |
| --- | --- |
| 2019 | 2.399577 |
| 2020 | 2.446467 |
| 2021 | 2.530677 |
| 2022 | 2.560606 |
| 2023 | 2.560606^a^ |

PPP, Purchase Power Parity.

^a^ Since USD-PPP rates were available only up to 2022, rates of 2022 were applied to convert cost incurred in 2023.

Source: PPPs and exchange rates (Edition 2023) [Internet]. 2024. Available from: https://www.oecd-ilibrary.org/content/data/154b256b-en.

**Supplemental Table 3** Regression model output: costs by sex, age, and disease type.

| **Coefficients** | **Estimate** | **Standard Error** | ***t* Statistic** | **p-value** |
| --- | --- | --- | --- | --- |
| Intercept | 376.55173 | 57.67335 | 6.529 | 0.0000000000676^a^ |
| Males | 15.08498 | 15.31090 | 0.985 | 0.325 |
| Age | 0.02168 | 0.25209 | 0.086 | 0.931 |
| Meningitis | 1233.88318 | 73.16106 | 17.0935415 | 0.0000000000000002^a^ |
| Pneumonia | 54.12635 | 57.65704 | 0.91258251 | 0.36147194 |
| Septicemia | 282.88862 | 56.96639 | 4.966 | 0.0000006888864^a^ |

Significance codes: ^a^ p < 0.001.
